# Urbanization Impacts Top Predators and Alters Biotic Interactions in Predator–Prey–Mutualistic Communities of Urban Dry Grasslands

**DOI:** 10.1002/ece3.70791

**Published:** 2025-01-11

**Authors:** Tanja M. Straka, Viktoriia Radchuk, Ingo Kowarik, Moritz von der Lippe, Sascha Buchholz

**Affiliations:** ^1^ Department of Ecology Technische Universität Berlin Berlin Germany; ^2^ Berlin‐Brandenburg Institute of Advanced Biodiversity Research (BBIB) Berlin Germany; ^3^ Freie Universität Berlin, Institute of Biology Berlin Germany; ^4^ Department of Ecological Dynamics Leibniz Institute for Zoo and Wildlife Research Berlin Germany; ^5^ University of Münster, Institute of Landscape Ecology Münster Germany

**Keywords:** biotic interactions, ecosystem services, multitrophic interactions, urban biodiversity, urban ecosystems, urban grassland, wild bees

## Abstract

Urbanization as a major driver of global change modifies biodiversity patterns and the abundance and interactions among species or functional species groups. For example, urbanization can negatively impact both predator–prey and mutualistic relationships. However, empirical studies on how urbanization modifies biotic, particularly multitrophic, interactions are still limited. In this study, we applied a framework focused on a predator–prey–mutualistic relationship involving communities of insect‐pollinated vascular plants, pollinators (bees and hoverflies), predatory spiders, and sand lizards as top predators to test (i) the effect of urbanization on abundance and species richness at different trophic levels and (ii) the effect of urbanization on the regulation of biotic interactions using correlations between species abundances as a proxy. By assessing 56 dry grassland patches in Berlin, Germany, we found that higher trophic levels (sand lizard abundance as well as predatory spider species richness and abundance) were significantly impacted by urbanization whereas pollinators were affected to a lesser degree (only abundance, but not species richness). In contrast, insect‐pollinated vascular plants were not impacted by urbanization. Path analyses revealed significant relationships in low‐urbanized areas. In these areas, we observed significant bottom‐up‐regulated mutualistic and predator–prey interactions (plants–pollinators, and pollinators–predatory spiders), as well as top‐down‐regulated predator–prey interactions (sand lizards–pollinators, and predatory spiders–pollinators). In contrast, no significant interactions were found in highly urbanized sites. Our results suggest that bottom‐up regulation is stronger than top‐down regulation in low‐urbanized areas. To our knowledge, this is the first study to examine the effects of urbanization on predator–prey–mutualistic interactions and to determine whether these interactions are regulated by bottom‐up or top‐down processes. These findings enhance our understanding of multitrophic interactions in urban environments and their associated ecosystem services, such as pollination, thereby supporting efforts in urban biodiversity conservation.

## Graphical abstract

We investigated multitrophic interactions involving insect‐pollinated plants, pollinators, spiders, and sand lizards in both highly urbanized and less urbanized areas of Berlin. Our findings indicate that urbanization significantly affects higher trophic levels and biotic interactions, with intertrophic relationships only observed in less urbanized sites.

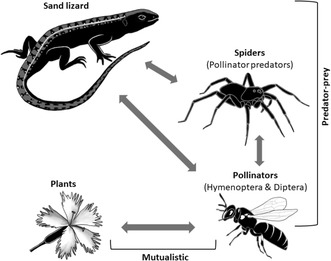

## 1 Introduction

The ongoing urban land expansion (Chen et al. 2020) is driving the global extinction crisis (IPBES 2019), threatening biodiversity in nonurban landscapes (McDonald et al. 2020) and modifying species assemblages and associated ecosystem services in cities as well (Elmqvist et al. 2013; Aronson et al. 2014). While urbanization impacts on biodiversity are increasingly understood, open questions remain unexplored (Knapp et al. 2021; Rega‐Brodsky, Aronson, et al. 2022), for example, on how different taxonomic groups respond to urbanization. Compared with their surroundings, some cities are richer in bird (Callaghan et al. 2019) and vascular plant species (Kühn, Brandl, and Klotz 2004) and can also harbor endangered species (Kühn, Brandl, and Klotz 2004; Ives et al. 2016; Soanes and Lentini 2019). The rates of plant population establishment and survival, however, differs across urban environments (Kowarik and von der Lippe 2018; Planchuelo, Kowarik, and von der Lippe 2020). Bee species generally perform better in urban settings compared to agricultural and rural areas (Baldock et al. 2015; Remmers and Frantzeskaki 2024), with cities strongly filtering for functional traits (Normandin et al. 2017; Buchholz and Egerer 2020). In contrast to bee species, butterfly species respond negatively to urbanization (Theodorou et al. 2020) as did nine taxa from aquatic, semiaquatic and terrestrial habitats, yet with pronounced differences among taxa (Piano et al. 2020). One insight from such studies is that urbanization effects vary significantly among taxa and urban environments.

Studies on biotic interactions in urban systems have received growing attention in the last years (Start, Barbour, and Bonner 2020; Rocha and Fellowes 2018, 2020; Theodorou 2022; Planillo et al. 2021). Empirical studies on how urbanization modifies multispecies and multitrophic interactions, however, remain limited in number (Rega‐Brodsky, Aronson, et al. 2022). However, this knowledge is crucial since biotic interactions such as mutualistic interactions or predator–prey relationships affect biodiversity and even communities themselves (Stachowicz 2001; Cavieres et al. 2014; Martin and Bonier 2018) and may support important ecosystem functions such as pollination, pest control (Barnes et al. 2020; Theodorou et al. 2020; Theodorou 2022), and soil multifunctionality (Schittko et al. 2022).

Earlier interest in how urbanization affects herbivory, and, in turn, pest control, inspired research (Turrini, Sanders, and Knop 2016) on trophic cascades, for example, the positive effects of predators on producers via negative effects on herbivores (Paine 1980). Similarly, mutualists are also affected by predators, parasitoids, competitors, and producers, and the indirect effects of trophic interactions on mutualism, such as plant–pollinator relations, have received increasing attention (Suttle 2003; Brechbühl, Kropf, and Bacher 2010; Gillespie and Adler 2013; Benoit and Kalisz 2020). For example, Suttle (2003) has shown that a predator spider species (*Misumenops schlingeri*), by preying on pollinators of the ox‐eye daisy (*Leucantheum vulgare*), decreases its seed production. Additionally, Benoit and Kalisz (2020) demonstrated in their review of predator effects on plant–pollinator interactions that predators can negatively influence pollinator visitation rates and the duration of pollinator visits to plants. This, in turn, can result in reduced pollen transfer and limited seed production. Despite the emerging consideration of the impacts that predators have on mutualistic relations, we know little about how urbanization affects such effects on mutualists. Although the impact of urbanization on pollination increasingly attracts more attention (Theodorou et al. 2020; Wenzel et al. 2020; Cohen et al. 2021; Theodorou 2022), the research is mainly conducted from the point of view of pollination service and thus investigates plant–pollinator interactions. How such plant–pollinator interactions are modified by urbanization when embedded in a larger network of predators (and their enemies) remains little studied (but see Gillespie and Adler 2013) despite its consequences for ecosystem services.

## 2 Regulation of Biotic Interactions in Urban Areas

**FIGURE 1:**
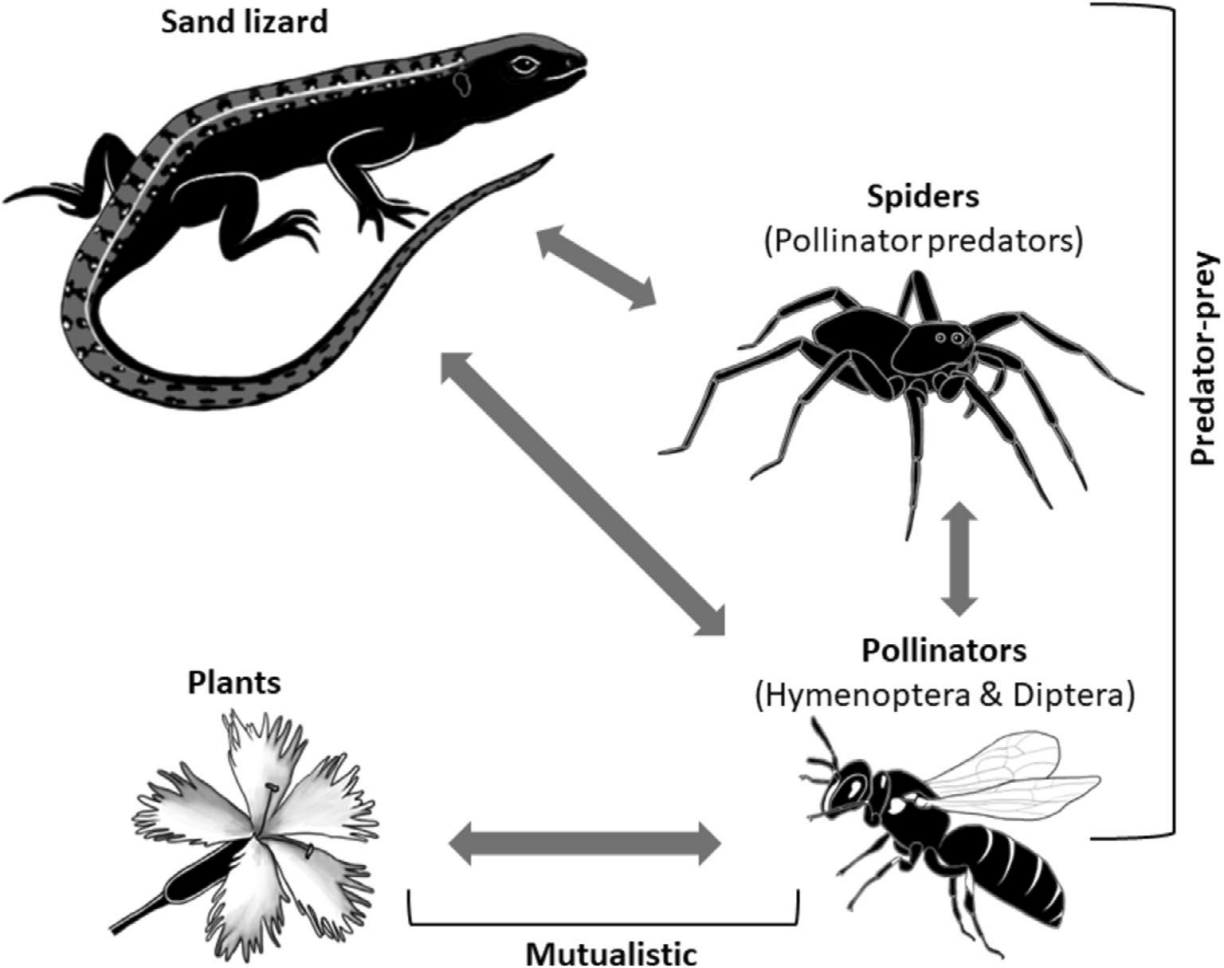
An *a priori* model showing expected interactions between insect‐pollinated vascular plants and pollinators (mutualistic) embedded in trophic interactions with spiders and sand lizards (predator–prey) that were tested in dry grassland of high and less urbanized areas. We refer to such interactions as predator–prey–mutualistic throughout the text.

Evidently, urbanization can influence and even disrupt predator–prey–mutualist interactions through altering the abundance and composition of communities (Start, Barbour, and Bonner 2020; Korányi et al. 2021). Scholars refer to some species, for instance, as urban tolerant, urban sensitive, or even synanthropic based on their response to urbanization and their ability to live or even thrive in the urban environment (McDonnell and Hahs 2015, Russo and Ancillotto 2015, Gathof et al. 2022). This taxon or even species‐specific response to urbanization influences the taxonomic and functional composition of urban biodiversity. Generally, high trophic‐level species (e.g., predators) tend to be more affected by heavily urbanized environments than low trophic‐level species (Faeth et al. 2005; Korányi et al. 2021). This is likely a result of the higher sensitivity of the former species to habitat fragmentation (Shochat et al. 2006) and environmental disturbances that arise from habitat alteration (Sorace and Gustin 2009; Turrini, Sanders, and Knop 2016). In contrast, plant species richness of both native and non‐native species, that is, primary producers, is suggested to increase in urban areas and mainly due to urban habitat heterogeneity (Kühn, Brandl, and Klotz 2004; Wania, Kühn, and Klotz 2006; Faeth, Bang, and Saari 2011). However, it is also influenced by the social environment and people's choices for certain plant species based on factors such as aesthetics or drought tolerance (Kendal, Williams, and Williams 2012). In contrast, mixed results are found for arthropod species that often occupy different trophic levels; for example, are both a predator and a prey simultaneously such as spiders (Figure 1) (Nyffeler 1999; Buschini et al. 2010; Mamou et al. 2016). In addition, as for other taxa, different arthropod species respond differently (negative or positive) to urbanization (Faeth, Bang, and Saari 2011; Fischer et al. 2016; Lowe et al. 2018; Korányi et al. 2021); making it even more difficult to predict how urbanization shapes predator–prey–mutualistic interactions in which arthropods are embedded. In this context, pollinating arthropods play a particular important role in both nontrophic mutualistic and trophic predator–prey networks (Theodorou et al. 2020). They, therefore, form a special interface between primary producers and consumers at higher levels (Schmalhofer 2001).

Studies of multitrophic systems often attempt to understand whether the system is regulated top‐down by a predominantly negative effect of predators on prey that cascades through the food web; or bottom‐up by the positive effects of producers on prey abundance (Power 1992; Miles et al. 2019; Barnes et al. 2020). Essentially, species from high trophic levels (e.g., predators) need lower trophic levels (e.g., herbivores they feed on) which also rely on primary producers (e.g., plants) to be able to persist in urban areas (Figure 1). The direction of regulation can be, among other factors, influenced by the abundance within each trophic level (Shochat 2004; Theodorou et al. 2020). For instance, an increased abundance of herbivores might be a result of the high abundance of primary producers (bottom‐up control), low pressure of predators (top‐down regulation), or a combination of both (Shochat 2004). In fact, many food webs are the result of the interplay of top‐down and bottom‐up forces (Power 1992). Bottom‐up control has been suggested to prevail in urban environments given that urban plant communities are often directly controlled by humans and act as a base for subsequent ecological and evolutionary processes (Faeth, Bang, and Saari 2011). The same direction of control applies for human‐altered food resources in urban areas, for example, when bird abundance is positively related to higher food supply in urban habitats (e.g., bird feeders, exotic vegetation, Marzluff 2001). Other studies showed how urbanization can limit top‐down control if one trophic level is limited in the urban environment (Turrini, Sanders, and Knop 2016) or how urbanization through, for example, the alteration of water availability or pollution can impact the abundance of primary producers (Miles et al. 2019). Understanding whether an urban ecosystem is bottom‐up or top‐down‐regulated is crucial for conservation measures that support species involved in trophic interactions that play key roles in ecosystem functions such as predation or pollination.

To address this issue, we used a system consisting of mutualists (i.e., insect‐pollinated vascular plants and pollinators) embedded in a food web of spiders that feed on pollinators (first‐order consumers) and the top‐predator sand lizard (*Lacerta agilis*), which could prey on both spiders and pollinators. We refer to communities that consist of predators, prey, pollinators, and plants as the “predator–prey–mutualistic system” henceforth (Figure 1). By investigating a predator–prey–mutualist system in low‐ and high‐urbanized dry grassland sites in the city of Berlin, we aimed to assess the following hypotheses:

*High trophic‐level communities (e.g., sand lizards and predatory spiders) in predator–prey–mutualistic systems are more sensitive to urbanization compared with low trophic‐level arthropods (e.g., pollinators) that benefit from primary producers (e.g., insect‐pollinated vascular plants). This will be expressed by a lower abundance and species richness of high‐ compared with low‐trophic level communities*.

*As a consequence, urbanization leads to bottom‐up controlled predator–prey–mutualistic systems involving pollinators*.

## 3 Materials and Methods

### 3.1 Study Sites

The study was carried out in Berlin, Germany, which covers an area of 892 km^2^ and counted 3.7 million citizens in 2017, the year of the field survey. The selected study sites were 56 dry grassland patches that were part of *CityScapeLab* Berlin, a permanent research infrastructure of the Berlin‐Brandenburg Institute of Advanced Biodiversity Research (BBIB) for urban biodiversity research (von der Lippe et al. 2020). These sites were selected based on a stratified random selection of dry grassland patches in the metropolitan area of Berlin along an urbanization gradient. As the basis for the random site selection, we choose all potential sandy dry grassland patches from the official Berlin biotope mapping (Senate Department for Urban Development and Housing, 2014). The patches were stratified according to their age, connectivity and an estimation of urbanity by the amount of impervious surface in a 500 m buffer around the patches. The variables age and connectivity were not used for this study. From each of the resulting strata, a similar number of patches were randomly selected for sampling. Sampling sites were then randomly placed within the selected patches as a 4 × 4 m plot with the research tool “random points” in QGIS version 2.18.0.

These sites allow a comparison of urban effects on a standardized model system (i.e., dry grassland) that broadly spans an urbanization gradient in Berlin. Grassland patches shared a combination of herbs and grass species, belong to the same vegetation type (i.e., Sedo‐Sclerantheatea), were mown once or twice a year, and were not irrigated, fertilized, or treated with herbicides. In consequence, they clearly differ from traditional short‐cut lawns in urban parks and are generally of conservation concern in Berlin as in many other cities. For further details on the study systems, see von der Lippe et al. (2020).

### 3.2 Quantifying Urbanization

The percentage of impervious surface (i.e., roads and buildings) as a well‐established proxy for urbanization (Arnold Jr and Gibbons 1996; Lu and Weng 2006) was calculated using a buffer of 500 m radius around each of the study sites using QGIS version 2.18.0. This buffer size was used in alignment with the original selection of these sites with the CityScapeLab to untangle urbanization effects on biodiversity (von der Lippe et al. 2020) and is a buffer size used in a similar multitrophic study (Korányi et al. 2021). To separate the dataset into sites with high and low urbanization for our analyses, we used the median value (12.70%) of the impervious surface of the 56 sites as the cutoff point and assigned sites > 12.70% impervious surface (*n* = 28 sites) as highly urbanized (ranging from 13.1% to 67.6%) and sites with < 12.70% impervious surface (*n* = 28 sites) as low‐urbanized sites (ranging from 3% to 12.7%). The primary difference between high‐ and low‐urbanized sites was consequently the level of impervious surfaces around the grassland sites which we used in our study as proxy for urbanization due to its correlation with factors such as human activity, light pollution, temperature, and street runoff (Arnold Jr and Gibbons 1996; Arnfield 2003; Hale et al. 2013).

### 3.3 Multitaxa Surveys

In our multitaxa survey, we collected data on insect‐pollinated vascular plants to assess mutualistic relationships with pollinators, as well as on sand lizards that feed on pollinators and spiders, and on spiders that prey on pollinators to assess predator–prey relationships. The abundance of insect‐pollinated vascular plants, pollinators (Hymenoptera and Diptera), spiders (pollinator–predatory spiders), and sand lizards were monitored from April to May and in July and September 2017; through the most favorable period and weather conditions for each biological group. Experienced and trained observers conducted the survey for each taxon at all sites.

For vascular plants, we recorded the identity and cover (in percentage according to Londo 1976) of all plant species within each 4 × 4 m randomly selected plots on site at each study site. For the analyses in this paper, we focused on insect‐pollinated plant species (according to BiolFlor: Klotz, Kühn, and Durka 2002; Kühn, Durka, and Klotz 2004) as a subset of total vascular plant species as these are relevant to pollinators. The most frequent plant species included *Berteroa incana* , *Centaurea stoebe* , *Cerastium semidecandrum* , *Potentilla argentea* , *Rumex acetosella* , and *Trifolium arvense* (Table S1).

Wild bees and hoverflies were sampled via colored triplet pan traps, which is a standard method to yield robust species inventories in urban settings (e.g., Buchholz and Egerer 2020). This method accounts for different color preferences between bee species and changing floral traits along an urbanization gradient (Cabon, Kracht, et al. 2022) and increases the efficiency of trap clusters (Nielsen et al. 2011) (plastic bowls with a radius of 7.25 cm and a depth of 5 cm depth; spray‐painted in bright yellow, white, and blue UV, Sparver Leuchtfarbe, Spray‐Color GmbH, Merzenich, Germany). The colored triplet pan traps were installed at the fringe of the study plots and were filled with 300 mL of 5% formaldehyde and one drop of detergent to mitigate surface tension and left on a 30 cm high wooden stick for three 72‐h sessions at either study site in May, July, and September 2017. The timing of the pan trap sampling reflects the most important periods for pollinator‐relevant flowering aspects in the analyzed dry grasslands (early summer, midsummer, late summer). Since seasonal patterns of bee abundances and species richness are more stable in urban environments compared with natural habitats in which floral resources can be a limitation in certain months of the year (Leong et al. 2016), we considered our sampling effort sufficient for our purpose. However, we also acknowledge that our sampling efforts did not take into account the potential variation in flower coverage surrounding our grassland areas, which may have affected pollinator communities. A total of 108 out of the 141 species belonged to the Apidae family and the remaining 33 species to the Syrphidae family (Table S1).

Other arthropods were sampled from May to July and September to October 2017 using uncovered pitfall traps (10 cm diameter, 16 cm depth plastic cups filled with 1% formalin‐detergent solution). A pitfall trap was placed at each of the four corners of the 4 × 4 m plot, and the traps were emptied every 4 weeks. Arthropods were identified to the species level, with the majority from the taxa Chilopoda, Coleoptera, and Araneae. Araneae were further distinguished into pollinator–predatory spiders based on biological data from Cardoso et al. (2011) and Nentwig et al. (2022); including the major families Araneidae, Linyphiidae, and Tetragnathidae, but also Philodromidae, Pisauridae, Salticidae, and Thomisidae (Table S1). Abundance was determined by adding the counts from the three surveys.

Lastly, the number of sand lizards (*Lacerta agilis*) at each site was counted in May, June, and July 2017 following standard methods (Bosbach and Weddeling 2005). The abundance of lizards was similar to that of arthropods determined by summing their counts from the three surveys. Counts took place on sunny days in the early morning or late afternoon and an observer walked along an 80 × 4 m transect at an average pace of 20 m/min.

### 3.4 Statistical Analysis

We used R version 4.3.3. (2024‐02‐29) for our analyses. To compare the abundances and species richness of pollinators (bees and hoverflies) and activity density and species richness of predators of pollinators (spiders) between high and low‐urbanized sites (H1), we used generalized linear models (GLMs) with negative binomial distributions to account for overdispersion. Insect‐pollinated vascular plants and sand lizards were also included in our analyses assessing the type of regulation (i.e., top‐down or bottom‐up) in our predator–prey–mutualistic models (H2) (Figure 1). However, since we focused on a single species of reptile predators, we were unable to focus on species richness and quantified only the difference in the abundance of sand lizards between areas with high and low urbanization levels using GLMs with negative binomial distributions. As for plants, we used the proportional coverage per plant species within a defined area summed up into a total coverage according to Londo (1976).

To test the type of regulation between sites with different levels of urbanization (H2), we assigned species to trophic levels based on their predicted biotic interactions: mutualistic interactions (plants and pollinators) and predator–prey (pollinators and predators of pollinators, as well as sand lizards as top predators since they are feeding on pollinator–predatory spiders and pollinators, Figure 1, Table S1). Although we did not directly measure interactions (e.g., pollinator visits), we used the correlation between plant and pollinator abundances as a proxy. We fitted structural equation models (SEMs) (Shipley 2016) to assess whether the data support a bottom‐up or top‐down regulation type. These models differ in the direction of the paths flowing from predictor to response variables, such that under the hypothesis of a bottom‐up regulated system the paths flow from lower trophic levels to higher ones, whereas the direction is reverse for the top‐down regulation (e.g., Kempel et al. 2023). We fitted the SEMs using the piecewiseSEM library (Lefcheck, Byrnes, and Grace 2016). For the bottom‐up model, we included the cover of plants as a predictor of pollinator abundance, whose abundance was used as a predictor of spider abundance. Finally, both spider abundance and pollinator abundance were used as predictors of lizard abundance (Figure 1). For the top‐down model, we include the abundance of lizards as a predictor of the abundance of spiders, the abundance of both spiders and lizards as predictors of pollinators' abundance, and finally, pollinator abundance as a predictor of the plants' abundance. Models with spider abundance and pollinator abundance as responses were fitted again using GLMs with negative binomial distribution, while the model with plant cover as response was fitted with a linear model because plant cover approximated normal distribution. Decisions on predictor and response variables per model were confirmed via tests of detected separation indicating causal relationships between variables (Shipley 2016) and the predictive power of the models was assessed using Nagelkerke *R* ^2^ (Nagelkerke 1991). To test to what extent the direction of regulation in networks is influenced by urbanization, we run “bottom‐up” and “top‐down” models separately for datasets with high‐urbanized sites (> 12.70% impervious surface; *n* = 28 sites) and with low‐urbanized sites (> 12.70% impervious surface, *n* = 28 sites). We used the Akaike information criterion (AIC, Burnham and Anderson, 2002) to compare the bottom‐up and the top‐down model for each urbanization level. We used a threshold value of delta AIC = 7 to consider that one model is supported by the data better than the other, the best model being the one with the lowest AIC value (Liddle 2007).

## 4 Results

In total, 129 individuals of sand lizards, 2274 individuals of pollinator–predatory spiders from 41 species and 2203 individuals of pollinators from 141 species were collected (Table S1). Lastly, a total of 179 insect‐pollinated vascular plants (excluding graminoids, as they are generally wind‐pollinated) flowering during the sampling period were sampled.

### 4.1 Abundances and Species Richness of Trophic Levels in High‐ and Low‐Urbanized Areas

**FIGURE 2:**
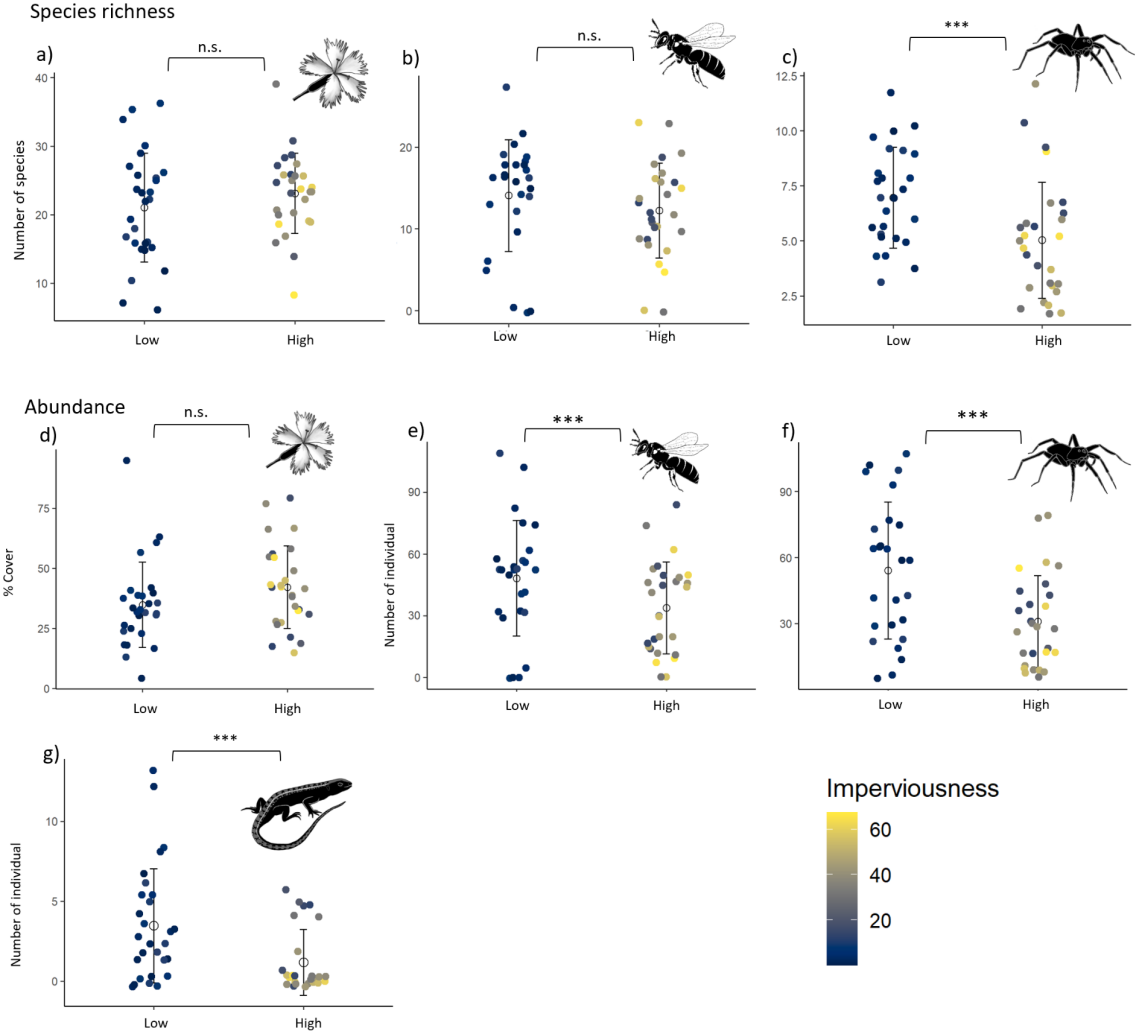
Comparison of species richness of (a) insect‐pollinated vascular plants, (b) pollinators, (c) predatory spiders and average abundance/cover of (d) insect‐pollinated vascular plants, (e) pollinators, (f) predatory spiders, and (g) sand lizards in low‐versus high‐urbanized areas. The white dot represents the mean, and the bar shows the standard deviation. The color gradient of the smaller points indicates the level of imperviousness at each site, ranging from dark blue (low imperviousness) to yellow (high imperviousness). Significant relationships are indicated with ***p < 0.001 and n.s. = not significant.

We found a significantly higher abundance of sand lizards and arthropods in low‐urbanized grassland areas compared to high‐urbanized grassland areas, while plant cover did not differ between sites (Table 1, Figure 2). The abundance of sand lizards was almost three times and pollinator–predatory spiders almost twice higher (1.7 times) in low‐ than in high‐urbanized sites. Slightly smaller but significant differences between high‐ and low‐urbanized sites were found for pollinators (1.5 times higher in low‐ than in high‐urbanized sites). When using imperviousness as a continuous variable, we did however not observe a significant effect on pollinator abundance (Table S2). For species richness, we found a significantly higher species richness for pollinator–predatory spiders in low‐ (1.5 times higher) than in high‐urbanized areas. The diversity of pollinator and plant species did not differ in high‐ than in low‐urbanized areas.

**TABLE 1:** Comparison of average (mean) and standard deviation (SD) of abundance/cover and species richness of trophic levels in high and low‐urbanized areas. Estimates (est.) and standard error (s.e.) show the effect sizes of low compared with high‐urbanized sites using generalized linear models (GLMs). For sand lizards, we show only abundance since it was only this reptile species sampled.

	Individuals (abundance)/cover (plants)			Species richness		
	High	Low		High	Low	
	Mean ± SD	Mean ± SD	Est. ± s.e., *p*	Mean ± SD	Mean ± SD	Est. ± s.e., *p*
Sand lizard	**1.2 ± 2.1**	**3.5 ± 3.6**	**1.84 ± 0.33**, **< 0.001**	—	—	—
Predatory spiders	**30.9 ± 20.8**	**54.2 ± 31.1**	**0.52 ± 0.05**, **< 0.001**	**5.0 ± 2.6**	**7.0 ± 2.3**	**0.43 ± 0.13**, **< 0.001**
Pollinators	**33.9 ± 22.4**	**48.2 ± 28.1**	**0.37 ± 0.05**, **< 0.001**	12.3 ± 5.1	14.1 ± 6.9	0.13 ± 0.08, 0.11
Plants	42.15 ± 17.2	34.76 ± 17.75	−8.73 ± 4.84, 0.08	23.1 ± 5.8	21.0 ± 7.9	−0.20 ± 1.97, 0.92

*Note:* Significant differences are marked in bold.

### 4.2 Impact of Urbanization on the Type of Regulation

#### 4.2.1 Low‐Urbanized Sites

**FIGURE 3:**
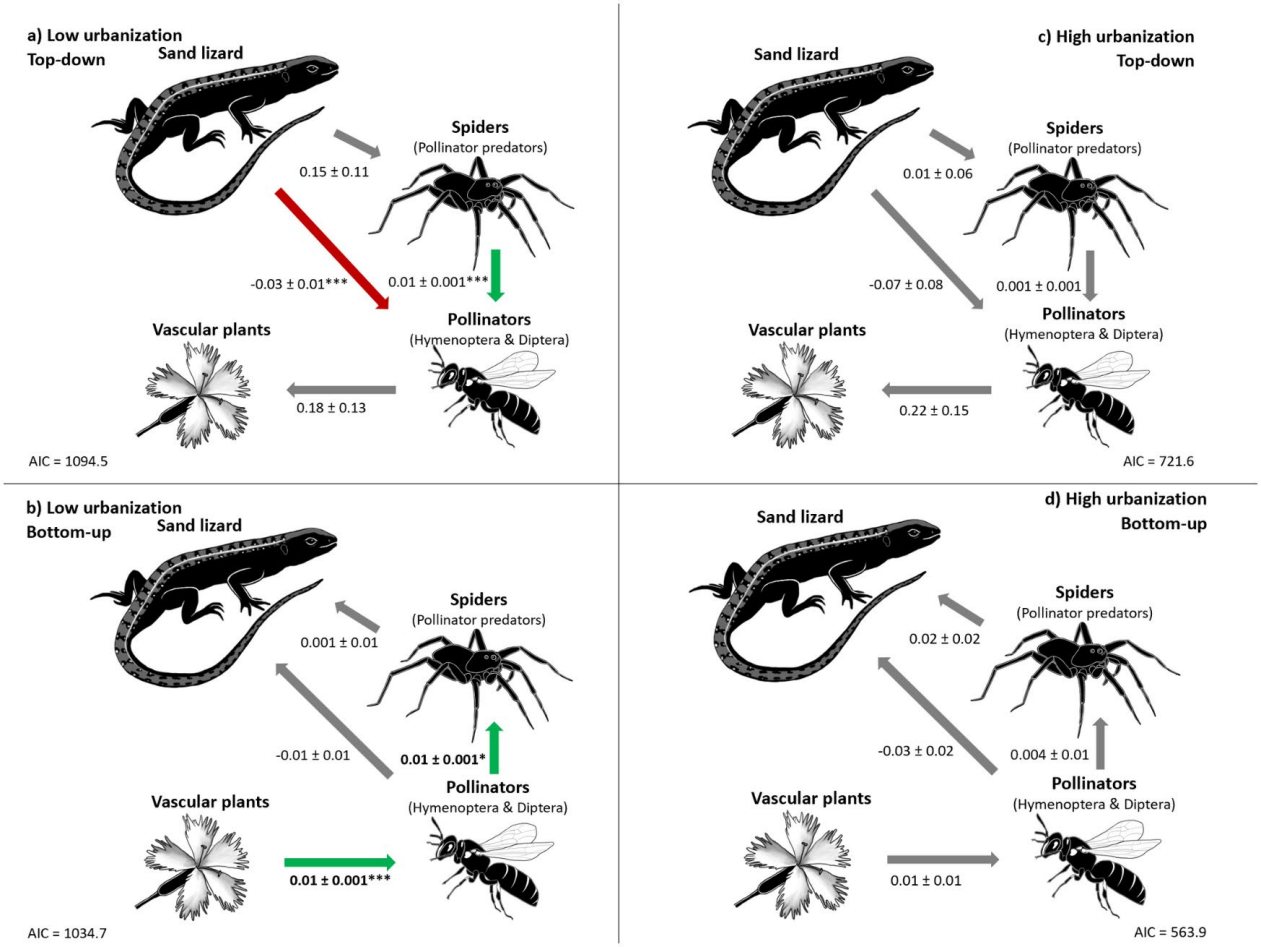
Relationships between the abundance of trophic levels using SEMs showing nonstandardized effect sizes and AIC of each model for (a) low urbanization—top‐down, (b) low urbanization—bottom‐up (somewhat best model for low urbanization), (c) high urbanization—top‐down, and (d) high urbanization—bottom‐up (somewhat best model for high urbanization). Interactions were confirmed through detection separation tests that indicated causal relationships between variables. Significant relationships are indicated with *p < 0.05, **p < 0.01, ***p < 0.001. Green indicates positive significant effects, red indicates negative significant effects, and gray indicates no significant effect.

Both bottom‐up and top‐down models resulted in a satisfactory fit to the data, as indicated by the nonsignificant *p* values from the chi‐squared and Fisher's *C* tests (bottom‐up: Fisher's *C* = 4.86, *p* value = 0.30, df = 4, chi‐squared = 2.56, *p* value = 0.28, df = 2; top‐down: Fisher's *C* = 3.72, *p* value = 0.45, df = 4, chi‐squared = 2.22, *p* value = 0.33, df = 2) (Figure 3). The data supported the bottom‐up model much more strongly (AIC = 1034.7) compared with the top‐down model (AIC = 1094.52) with an AIC∆ of 59, 87. According to the bottom‐up regulation model, we found a significant positive effect of plant cover on pollinator abundance and pollinator abundance on spider abundance (0.01 ± 0.001, *p* < 0.001 and 0.01 ± 0.001, *p* < 0.001, respectively). In contrast, the other path coefficients were nonsignificant. Nagelkerke *R* ^2^ values for the bottom‐up model were low for lizards (0.00), with a higher amount of variance explained for predatory spiders (0.22) and pollinators (0.65). Although less supported, there were two significant effects in the top‐down model. A higher abundance of sand lizards negatively affected the abundance of pollinators (−0.03 ± 0.01, *p* < 0.001) that was positively associated to a small extent with the abundance of spiders (0.001 ± 0.001, *p* < 0.01). The remaining path coefficients were nonsignificant. Nagelkerke *R* ^2^ values for the top‐down model were high for pollinators (0.93) but low for two other models: predatory spiders (0.00) and insect‐pollinated plants (0.07).

#### 4.2.2 High‐Urbanized Sites

Similarly, here, both bottom‐up and top‐down models resulted in a satisfactory fit to the data (bottom‐up: Fisher's *C* = 4.73, *p* value = 0.32, df = 4; Chi‐squared = 2.08, *p* value = 0.35, df = 2; top‐down: Fisher's *C* = 6.72, *p* value = 0.15, df = 4; chi‐squared = 4.28, *p* value = 0.12, df = 2) (Figure 3). The bottom‐up model was again better supported than the top‐down model according to the AIC (AIC for the bottom‐up: 563.86, and for the top‐down model: 721.62). We found no significant path coefficients in the bottom‐up and top‐down models at high‐urbanized sites. The Nagelkerke *R* ^2^ values for the models fitted to the high‐urbanized sites were much lower compared with those fitted to the low‐urbanized sites. Nagelkerke's *R* ^2^ values in the bottom‐up model were 0.07 for lizards, 0.02 for predatory spiders, and 0.09 for pollinators. Similarly, Nagelkerke *R* ^2^ values were low in the top‐down model: for predatory spiders (0.00), pollinators (0.05), and insect‐pollinated plants (0.08).

## 5 Discussion

Despite the widely known effect of urbanization on biodiversity, how urbanization modifies multispecies and multitrophic interactions has been still largely understudied (Rega‐Brodsky, Aronson, et al. 2022). Here, we used a “predator–prey‐mutualist” model system to test not only whether urbanization impacts high trophic‐level taxa but also the direction of community regulation (top‐down vs. bottom‐up). Our results indicated that the abundance and species richness of high trophic level arthropods and the abundance of sand lizards as top predators were significantly lower in high‐ than in low‐urbanized areas; indicating their sensitivity to urbanization and supporting our first hypothesis. Furthermore, we found stronger support for bottom‐up controlled regulations in both low‐ and high‐urbanized areas. However, significant path coefficients were only found in low‐urbanized areas. Consequently, we cannot clearly confirm our second hypothesis.

### 5.1 Abundance and Species Richness at High‐ and Low‐Urbanized Sites

The abundance and species richness of high trophic predatory spiders were significantly lower in high‐ than in low‐urbanized areas. This confirms our first hypothesis (H1) regarding the higher sensitivity of high trophic levels to urbanization (Shochat et al. 2006; Rocha and Fellowes 2020; Korányi et al. 2021). Sand lizards and predatory spiders were more abundant in low‐ than in high‐urbanized sites, with predatory spiders also higher in species richness.

Factors such as the presence of dogs (Weston and Stankowich 2014) and the absence of railway tracks as suitable dispersal corridors (Lucas, de Carvalho, and Grilo 2017) are known to have negative impacts on sand lizard populations in urban environments. Although sand lizards may have a certain tolerance to urbanization (Becker and Buchholz 2016) and seem to tolerate dog walking in urban dry grasslands in Berlin (Buchholz et al. 2021), we show that at least in smaller populations living on small habitat islands in the urban matrix, unspecified urban pressures appear to result in declining abundances.

Similarly, different urban pressures may have led to the lower abundance and species richness of predatory spiders in highly urbanized compared to low‐urbanized areas. In Rennes, France, for example, certain spider species, particularly large and heat‐sensitive species, responded negatively to the urban heat island effect, which increased with urbanization, while habitat complexity decreased in turn (Cabon et al. 2024). Since mowing frequency impacts arthropod communities (Proske, Lokatis, and Rolff 2022) and predatory spiders are sensitive to mowing (Stański et al. 2024) temporal variation in the mowing regime across study sites may have influenced the spider communities. Furthermore, the grassland areas are also subject to varying degrees of biotic disturbances, such as from wild boars (Cabon, Bùi, et al. 2022). However, some spider species or even families may not be as negatively affected by urbanization as others, as found for crab spider communities (Araneae: Thomisidae) which are very effective hunters in the herb layer and on flowers (Reader et al. 2006). On average, we found in our sample 17.1 species from the Thomisidae family in high‐urbanized areas and 17.7 species in low‐urbanized areas. Studies from other geographic regions using different sampling methods, such as vacuum sampling of vegetation in Argentina, also found that urbanization did not impact crab spider communities (Argañaraz and Gleiser 2017).

In contrast to higher trophic levels, the pollinator species richness was similar in low‐urbanized and high‐urbanized grassland plots. This finding reinforces the role of cities in hosting species‐rich pollinator communities, as demonstrated in the case of wild bees (Hall et al. 2017) and supports our first hypothesis. Among these pollinators, we identified 10 different species of bumblebees (genus *Bombus*) in our sample (Table S1) and, in particular, *Bombus lapidaries* have been found frequently in urban areas in Berlin (Gathof et al. 2022). Nevertheless, this does not imply that pollinators are not affected by urbanization. For instance, Gathof et al. (2022) observed that while *Bombus lapidarius* and *Lassioglossum morio* thrived in highly urbanized areas, *Andrena subopaca* was more prevalent in less urbanized regions of Berlin, Germany.

Similarly, there were no significant differences in species richness of insect‐pollinated plants in high versus low‐urbanized areas, also reinforcing the role of cities in hosting species‐rich and diverse plant assemblages (Kühn, Brandl, and Klotz 2004; Wania, Kühn, and Klotz 2006). This also holds for grassland at both near‐natural and anthropogenic sites in Berlin (Planchuelo, von der Lippe, and Kowarik 2019). Although plant species richness supports ecosystem functioning in terms of above‐ground productivity (Onandia et al. 2019) and below‐ground productivity (Schittko et al. 2022), similar high plant species richness and coverage, as well as pollinator richness and abundance in high‐urbanized areas were not associated with similar abundant predator communities within these biotic communities in our study system. In subsamples of our grassland plots, it has been shown that dogs (Buchholz et al. 2021), wild boars (Cabon, Kracht, et al. 2022), and heat island effects (Christmann et al. 2023) lead to responses in selected taxa. Since we were unable to collect the corresponding parameters for all areas, they were not included in the analysis.

### 5.2 Regulations of Communities in Areas With High and Low Urbanization

Our results suggest that bottom‐up regulation prevails in our predator–prey–mutualistic study system. However, this conclusion is not straightforward. Although the bottom‐up regulation model was better supported in high‐urbanized sites, we did not find any significant path coefficient in the models in high‐urbanized areas. Therefore, our findings do not clearly support our hypothesis (H2) that bottom‐up regulation should prevail in urbanized areas. This hypothesis was based on the idea that management can make spatially and temporally resources or primary producers more continuously available and thus mitigate the negative effect of urbanization on high trophic levels (Faeth et al. 2005; Shochat, Lerman, and Fernández‐Juricic 2010).

One possible explanation is that urbanization can disrupt predator–prey‐mutualist interactions by altering the abundance and composition of communities, as suggested by earlier studies (Start, Barbour, and Bonner 2020; Korányi et al. 2021), but also through the possible plant stress hypothesis (i.e., negative effect on primary producers through e.g., water stress, elevated temperature, pollution) (Miles et al. 2019). However, we did not find evidence that the abundance of lower trophic levels (plants and pollinators) was impacted by urbanization. Consequently, other factors such as higher air pollutant levels in high‐urbanized areas, though not measured in our study, are known to reduce insect‐mediated pollination services (Ryalls et al. 2022). This may explain the nonsignificant relationship between plants and pollinators in our high‐urbanized sites. Alternatively, pollinators are quite mobile and may feed on other flowering resources beyond those available in our grassland plots.

In areas with low urbanization levels, we found significant mutualistic relationships between insect‐pollinated vascular plants and pollinators, as well as predator–prey relationships between pollinators and predatory spiders in the bottom‐up‐regulated model. Although we did not measure pollinator visits directly and instead used species co‐occurrence as a proxy for plant–pollinator interactions, our findings are consistent with those of Ebeling et al. (2008), who observed a positive relationship between flowering plant cover and pollinator visits in their study on previously cultivated fields, rather than urban settings. This highlights the potential disruption of plant–pollinator interactions by other anthropogenic stressors in highly urbanized areas, as well as the critical role of plants as primary producers. However, our results somewhat contradict Theodorou et al. (2020), who demonstrated that pollination services (assessed with bumble bees) remain high in high‐urbanized areas. Our study contributes to the existing knowledge on plant–pollinator interactions at the community level: our results demonstrate that plant–pollinator interactions persist in low‐urbanized sites but are negatively impacted by urbanization when assessed at the community level. In the top‐down regulated model in low‐urbanized areas, sand lizards negatively affected pollinators, while the presence of predatory spiders positively affected pollinators. This observation may seem counterintuitive, as in the bottom‐up model, more pollinators were suggested to lead to more spiders which is a typical bottom‐up effect (i.e., an increase in prey abundance influences the predator abundance). Therefore, one would expect that more predatory spiders would reduce pollinator abundance. However, our model suggested the opposite: an increase in predatory spiders also leads to more pollinators. This may indicate indirect interactions. For instance, spiders may reduce the abundance of other arthropods that compete with pollinators for resources (e.g., herbivores that feed on insect‐pollinated vascular plants) which we did not account for in our models. Another possible explanation is that both spiders and pollinators are part of the diet of sand lizards (Blanke and Fearnley 2015). The presence of predatory spiders likely provides sand lizards with an alternative food source, thereby reducing the predation pressure on pollinators. In other words, sand lizards may shift their feeding behavior when spiders are abundant, leading to reduced predation on pollinators. Consequently, if sand lizards primarily feed on spiders when they are abundant, it could create a balance where spider abundance is regulated, preventing them from overpredation on pollinators. However, experimental designs would be needed to better understand the nuances of these interactions.

There are several caveats to our study. First, the cutoff point for defining high and low urbanization was relatively small, and we recognize that different findings might emerge with a higher cutoff point. However, due to variations between cities, there is no universally defined threshold for high or low urbanization (Hahs 2024). Consequently, we used the median as a threshold that evenly divided our dataset. Second, we did not assess pollinator visits but used a correlative approach and the co‐occurrence of species as a proxy of plant–pollinator interactions. Third, we did not analyze the regulation mechanisms by season as this was beyond the scope of our sampling efforts. However, future studies could focus on more intensive samplings over time to investigate seasonal effects. Fourth, future studies could benefit from additional sampling methods, such as beating trays or sweep nets, to better assess the abundance of herb‐dwelling spiders that prey on pollinators. Fifth, we did not include other organisms, such as herbivores, in our analyses, though they likely had a significant influence on the relationships between the trophic communities. Future research could incorporate herbivores into studies on predator–prey–mutualistic interactions to provide a more comprehensive understanding of their role in these interactions. Lastly, the timing of sampling relative to mowing events was not explicitly defined, which may have influenced our results and should be considered in future studies (Proske, Lokatis, and Rolff 2022).

## 6 Conclusions

Multitrophic interactions in urban ecosystems are intricate and under‐researched, despite their importance for biodiversity conservation and the sustainability of ecosystem services like pollination and pest regulation. Our study adds to this body of knowledge by examining predator–prey–mutualistic interactions among insect‐pollinated plants, pollinators, predatory spiders, and sand lizards across urbanization gradients. We observed a decline in top predators (sand lizards) and predatory spiders in highly urbanized areas while lower and no impact of urbanization on pollinators and insect‐pollinated plants, respectively. This confirms that urbanization impacts particularly higher trophic levels. Additionally, our study suggests that these predator–prey–mutualistic relationships are regulated by bottom‐up processes, indicating a primary dependence on resource availability. Consequently, conservation strategies in urban areas could benefit from prioritizing the enhancement of primary producers to support these predator–prey–mutualistic interactions with pollinators since this could also indirectly support higher trophic levels. Further research gaps remain in investigating bottom‐up and top‐down regulations across diverse biotic interactions such as competition, predation, parasitism, and mutualism. Addressing these gaps is essential for developing targeted conservation strategies that protect biodiversity, maintain ecosystem functions, and support ecosystem services.

## Author Contributions

**Tanja M. Straka:** conceptualization (supporting), formal analysis (lead), visualization (lead), writing – original draft (lead). **Viktoriia Radchuk:** conceptualization (supporting), formal analysis (supporting), visualization (supporting), writing – original draft (supporting). **Ingo Kowarik:** conceptualization (lead), funding acquisition (lead), writing – review and editing (equal). **Moritz von der Lippe:** conceptualization (lead), funding acquisition (lead), investigation (supporting), methodology (supporting), supervision (supporting), writing – review and editing (equal). **Sascha Buchholz:** conceptualization (lead), investigation (lead), methodology (lead), supervision (lead), writing – review and editing (equal).

## Conflicts of Interest

The authors declare no conflicts of interest.

## Supporting information


Data S1.

## Data Availability

The data supporting the results can be found in the Supporting Information.
