# Supplementary material for: Urbanization Impacts Top Predators and Alters Biotic Interactions in Predator–Prey–Mutualistic Communities of Urban Dry Grasslands
Source: Ecol Evol. 2025 Jan 11;15(1):e70791. doi: 10.1002/ece3.70791 (PMC11724209; doi:10.1002/ece3.70791)
Supplement: Supplementary file 1 — Data S1. [file ECE3-15-e70791-s001.zip › Suppl.Material.docx]

***Supplementary Material***

Title: Urbanization impacts top predators and alters biotic interactions in predator-prey-mutualistic communities of urban dry grasslands

Authors: Tanja M. Straka, Viktoriia Radchuk, Ingo Kowarik, Moritz von der Lippe, Sascha Buchholz

Table S1: Plant, pollinator and predator species used to investigate community compositions in high and low urbanized areas

| ***#*** | ***Insect-pollinated vascular plants*** | ***Pollinators*** | ***Predatory spiders*** |
| --- | --- | --- | --- |
| 1 | *Achillea millefolium* | *Andrena alfkenella* | *Argiope bruennichi* |
| 2 | *Agrimonia eupatoria* | *Andrena argentata* | *Aelurillus v-insignitus* |
| 3 | *Ajuga genevensis* | *Andrena barbilabris* | *Attulus distinguendus* |
| 4 | *Anchusa officinalis* | *Andrena bimaculate* | *Cercidia prominens* |
| 5 | *Anthriscus caucalis* | *Andrena cineraria* | *Coriarachne depressa* |
| 6 | *Arabidopsis arenosa* subsp. *arenosa* | *Andrena denticulate* | *Evarcha arcuate* |
| 7 | *Arabidopsis thaliana* | *Andrena dorsata* | *Evarcha falcata* |
| 8 | *Arenaria serpyllifolia* | *Andrena flavipes* | *Heliophanus flavipes* |
| 9 | *Armeria maritima* subsp. *elongata* | *Andrena haemorrhoa* | *Hypsosinga albovittata* |
| 10 | *Artemisia campestris* | *Andrena helvola* | *Linyphia hortensis* |
| 11 | *Artemisia vulgaris* | *Andrena nigroaenea* | *Linyphia triangularis* |
| 12 | *Berteroa incana* | *Andrena nigrospina* | *Mangora acalypha* |
| 13 | *Campanula rapunculus* | *Andrena nitida* | *Misumena vatia* |
| 14 | *Capsella bursa-pastoris* | *Andrena semilaevis* | *Neriene clathrate* |
| 15 | *Cardamine hirsuta* | *Andrena subopaca* | *Ozyptila atomaria* |
| 16 | *Carex arenaria* | *Andrena tibialis* | *Ozyptila claveata* |
| 17 | *Centaurea jacea* | *Anthidium punctatum* | *Ozyptila praticola* |
| 18 | *Centaurea stoebe* | *Anthophora furcate* | *Ozyptila scabricula* |
| 19 | *Cerastium arvense* | *Apis mellifera* | *Ozyptila trux* |
| 20 | *Cerastium glomeratum* | *Bombus bohemicus* | *Pellenes tripunctatus* |
| 21 | *Cerastium glutinosum* | *Bombus hortorum* | *Philodromus cespitum* |
| 22 | *Cerastium holosteoides* | *Bombus hypnorum* | *Philodromus collinus* |
| 23 | *Cerastium semidecandrum* | *Bombus lapidaries* | *Phlegra fasciata* |
| 24 | *Chenopodium album* | *Bombus lucorum* | *Phylloneta impressa* |
| 25 | *Chondrilla juncea* | *Bombus pascuorum* | *Pisaura mirabilis* |
| 26 | *Cirsium arvense* | *Bombus pratorum* | *Sittiflor zimmermanni* |
| 27 | *Cirsium vulgare* | *Bombus ruderarius* | *Tetragnatha extensa* |
| 28 | *Clematis vitalba* | *Bombus rupestris* | *Thanatus arenarius* |
| 29 | *Convolvulus arvensis* | *Bombus soroeensis* | *Thomisus onustus* |
| 30 | *Conyza canadensis* | *Bombus sylvarum* | *Tibellus oblongus* |
| 31 | *Crataegus monogyna* | *Bombus sylvestris* | *Xysticus bifasciatus* |
| 32 | *Crepis capillaris* | *Bombus terrestris* | *Xysticus cristatus* |
| 33 | *Cynoglossum officinale* | *Bombus vestalis* | *Xysticus erraticus* |
| 34 | *Cytisus scoparius* | *Cheilosia vernalis agg.* | *Xysticus kochi* |
| 35 | *Dactylis glomerata* | *Chelostoma rapunculi* | *Xysticus lanio* |
| 36 | *Daucus carota* | *Chrysotoxum bicinctum* | *Xysticus luctator* |
| 37 | *Dianthus carthusianorum* | *Chrysotoxum festivum* | *Xysticus luctuosus* |
| 38 | *Dianthus deltoides* | *Chrysotoxum veralli* | *Xysticus ninnii* |
| 39 | *Dianthus giganteus* | *Coelioxys conica* | *Xysticus robustus* |
| 40 | *Dipsacus fullonum* | *Dasypoda hirtipes* | *Xysticus striatipes* |
| 41 | *Draba verna* | *Dasyrphus albostriatus* | *Xysticus ulmi* |
| 42 | *Echium vulgare* | *Didea intermedia* |  |
| 43 | *Erigeron acris* | *Episyrphus balteatus* |  |
| 44 | *Erigeron annuus* | *Eristalinus sepulchralis* |  |
| 45 | *Erodium cicutarium* | *Eristalis abusiva* |  |
| 46 | *Euonymus europaea* | *Eristalis arbustorum* |  |
| 47 | *Euphorbia cyparissias* | *Eristalis intricaria* |  |
| 48 | *Ficaria verna* | *Eristalis nemorum* |  |
| 49 | *Filago arvensis* | *Eristalis similis* |  |
| 50 | *Gagea pratensis* | *Eristalis tenax* |  |
| 51 | *Galium aparine* | *Ferdinandea cuprea* |  |
| 52 | *Galium boreale* | *Halictus confuses* |  |
| 53 | *Galium mollugo* | *Halictus rubicundus* |  |
| 54 | *Galium verum* | *Halictus sexcinctus* |  |
| 55 | *Geranium molle* | *Halictus subauratus* |  |
| 56 | *Geranium pusillum* | *Halictus submediterraneus* |  |
| 57 | *Geranium pyrenaicum* | *Halictus tumulorum* |  |
| 58 | *Geranium robertianum* | *Helophilus pendulus* |  |
| 59 | *Glechoma hederacea* | *Helophilus trivittatus* |  |
| 60 | *Gypsophila paniculata* | *Heriades crenulatus* |  |
| 61 | *Helichrysum arenarium* | *Heriades truncorum* |  |
| 62 | *Herniaria glabra* | *Hoplitis adunca* |  |
| 63 | *Hieracium bauhinii* | *Hoplitis anthocopoides* |  |
| 64 | *Hieracium pilosella* | *Hoplitis leucomelana* |  |
| 65 | *Hieracium umbellatum* | *Hylaeus angustatus* |  |
| 66 | *Holosteum umbellatum* | *Hylaeus brevicornis* |  |
| 67 | *Hypericum perforatum* | *Hylaeus communis* |  |
| 68 | *Hypochaeris radicata* | *Hylaeus confuses* |  |
| 69 | *Jasione montana* | *Hylaeus dilatatus* |  |
| 70 | *Lamium purpureum* | *Hylaeus gredleri* |  |
| 71 | *Lapsana communis* | *Hylaeus hyalinatus* |  |
| 72 | *Leucanthemum vulgare* agg. | *Hylaeus punctatus* |  |
| 73 | *Linaria vulgaris* | *Hylaeus signatus* |  |
| 74 | *Lotus corniculatus* | *Hylaeus sinuatus* |  |
| 75 | *Luzula campestris* | *Lasioglossum ageratum* |  |
| 76 | *Luzula multiflora* | *Lasioglossum albipes* |  |
| 77 | *Malva moschata* | *Lasioglossum brevicorne* |  |
| 78 | *Medicago lupulina* | *Lasioglossum calceatum* |  |
| 79 | *Medicago minima* | *Lasioglossum fulvicorne* |  |
| 80 | *Medicago* x *varia* | *Lasioglossum laticeps* |  |
| 81 | *Melampyrum pratense* | *Lasioglossum leucopus* |  |
| 82 | *Melilotus officinalis* | *Lasioglossum leucozonium* |  |
| 83 | *Moehringia trinervia* | *Lasioglossum lucidulum* |  |
| 84 | *Myosotis arvensis* | *Lasioglossum monstrificum* |  |
| 85 | *Myosotis ramosissima* | *Lasioglossum morio* |  |
| 86 | *Myosotis stricta* | *Lasioglossum pauxillum* |  |
| 87 | *Oenothera biennis* agg. | *Lasioglossum quadrinotatum* |  |
| 88 | *Ononis repens* | *Lasioglossum setulosum* |  |
| 89 | *Papaver argemone* | *Lasioglossum sexnotatum* |  |
| 90 | *Papaver dubium* | *Lasioglossum sexstrigatum* |  |
| 91 | *Papaver* spec. | *Lasioglossum* spec. |  |
| 92 | *Petrorhagia prolifera* | *Lasioglossum villosulum* |  |
| 93 | *Petrorhagia saxifraga* | *Macropis europaea* |  |
| 94 | *Peucedanum oreoselinum* | *Megachile circumcincta* |  |
| 95 | *Picris hieracioides* | *Megachile ligniseca* |  |
| 96 | *Pimpinella saxifraga* agg. | *Megachile maritima* |  |
| 97 | *Plantago arenaria* | *Megachile rotundata* |  |
| 98 | *Plantago lanceolata* | *Megachile versicolor* |  |
| 99 | *Plantago major* | *Megachile willughbiella* |  |
| 100 | *Polygonatum odoratum* | *Melanostoma mellinum* |  |
| 101 | *Polygonum aviculare* | *Melanostoma scalare* |  |
| 102 | *Portulaca oleracea* | *Melecta albifrons* |  |
| 103 | *Potentilla argentea* | *Melitta leporine* |  |
| 104 | *Potentilla incana* | *Merodon equestris* |  |
| 105 | *Potentilla reptans* | *Myathropa florea* |  |
| 106 | *Potentilla verna* | *Nomada alboguttata* |  |
| 107 | *Prunus spinosa* | *Nomada flavoguttata* |  |
| 108 | *Ranunculus acris* | *Nomada flavopicta* |  |
| 109 | *Ranunculus bulbosus* | *Nomada lathburiana* |  |
| 110 | *Rosa canina* agg. | *Nomada moeschleri* |  |
| 111 | *Rubus caesius* | *Nomada panzer* |  |
| 112 | *Rubus* sect. *Rubus* | *Nomada ruficornis* |  |
| 113 | *Rumex acetosella* | *Osmia bicolor* |  |
| 114 | *Rumex thyrsiflorus* | *Osmia bicornis* |  |
| 115 | *Salvia pratensis* | *Osmia brevicornis* |  |
| 116 | *Saponaria officinalis* | *Osmia mustelina* |  |
| 117 | *Saxifraga tridactylites* | *Panurgus calcaratus* |  |
| 118 | *Scleranthus annuus* agg. | *Pipiza festiva* |  |
| 119 | *Scleranthus perennis* | *Scaeva selenitica* |  |
| 120 | *Scorzoneroides autumnalis* | *Sericomyia silentis* |  |
| 121 | *Securigera varia* | *Sphaerophoria batava* |  |
| 122 | *Sedum acre* | *Sphaerophoria scripta* |  |
| 123 | *Sedum album* | *Sphaerophoria* spec. |  |
| 124 | *Sedum maximum* | *Sphecodes albilabris* |  |
| 125 | *Sedum rupestre* | *Sphecodes crassus* |  |
| 126 | *Sedum sexangulare* | *Sphecodes ferruginatus* |  |
| 127 | *Senecio inaequidens* | *Sphecodes longulus* |  |
| 128 | *Senecio jacobaea* | *Sphecodes miniatus* |  |
| 129 | *Senecio vernalis* | *Sphecodes niger* |  |
| 130 | *Silene conica* | *Sphecodes pellucidus* |  |
| 131 | *Silene latifolia* subsp. *alba* | *Sphecodes puncticeps* |  |
| 132 | *Silene vulgaris* | *Spheocdes monilicornis* |  |
| 133 | *Sisymbrium loeselii* | *Stelis breviuscula* |  |
| 134 | *Solanum nigrum* | *Syritta pipiens* |  |
| 135 | *Solidago canadensis* | *Syrphus ribesii* |  |
| 136 | *Spergula morisonii* | *Syrphus* spec. |  |
| 137 | *Spergularia rubra* | *Syrphus vitripennis* |  |
| 138 | *Stellaria graminea* | *Systropha curvicornis* |  |
| 139 | *Stellaria media* | *Tetraloniella dentata* |  |
| 140 | *Stellaria pallida* | *Volucella inanis* |  |
| 141 | *Tanacetum vulgare* | *Xylota segnis* |  |
| 142 | *Taraxacum* sect. *Erythrosperma* |  |  |
| 143 | *Taraxacum* sect. *Ruderalia* |  |  |
| 144 | *Teesdalia nudicaulis* |  |  |
| 145 | *Thymus pulegioides* |  |  |
| 146 | *Tragopogon dubius* |  |  |
| 147 | *Tragopogon pratensis* |  |  |
| 148 | *Tragopogon* spec. |  |  |
| 149 | *Trifolium arvense* |  |  |
| 150 | *Trifolium campestre* |  |  |
| 151 | *Trifolium dubium* |  |  |
| 152 | *Trifolium pratense* |  |  |
| 153 | *Trifolium repens* |  |  |
| 154 | *Trifolium striatum* |  |  |
| 155 | *Turritis glabra* |  |  |
| 156 | *Verbascum lychnitis* |  |  |
| 157 | *Verbascum nigrum* |  |  |
| 158 | *Veronica arvensis* |  |  |
| 159 | *Veronica chamaedrys* |  |  |
| 160 | *Veronica officinalis* |  |  |
| 161 | *Veronica prostrata* |  |  |
| 162 | *Veronica sublobata* |  |  |
| 163 | *Vicia angustifolia* |  |  |
| 164 | *Vicia cracca* |  |  |
| 165 | *Vicia hirsuta* |  |  |
| 166 | *Vicia lathyroides* |  |  |
| 167 | *Vicia sepium* |  |  |
| 168 | *Vicia tetrasperma* |  |  |
| 169 | *Vicia villosa* |  |  |
| 170 | *Viola arvensis* |  |  |
| 171 | *Viola canina* |  |  |
| 172 | *Viola suavis* |  |  |
| 173 | *Viola tricolor* |  |  |

Table S2: Effect of imperviousness (within 500m buffer) on bundance and species richness of communities. Generalized linear models with negative binomial distribution were applied, except for the coverage of insect-pollinated plants.

| **Abundance** | **Effect size ± S.E.** |
| --- | --- |
| Insect-pollinated plants (coverage) | 0.15 ± 0.12 |
| Pollinators | -0.01 ± 0.01 |
| **Predatory spiders** | **-0.01 ± 0.01**** |
| **Lizards** | **-0.06 ± 0.01***** |
|  |  |
| **Species richness** | **Effect size ± S.E.** |
| Insect-pollinated plants | 0.001 ± 0.05 |
| Pollinators | -0.004 ± 0.004 |
| **Predatory spiders** | **-0.009 ± 0.003**** |
