# Supplementary figures and images for: Urbanization Impacts Top Predators and Alters Biotic Interactions in Predator–Prey–Mutualistic Communities of Urban Dry Grasslands
Source: Ecol Evol. 2025 Jan 11;15(1):e70791. doi: 10.1002/ece3.70791 (PMC11724209; doi:10.1002/ece3.70791)

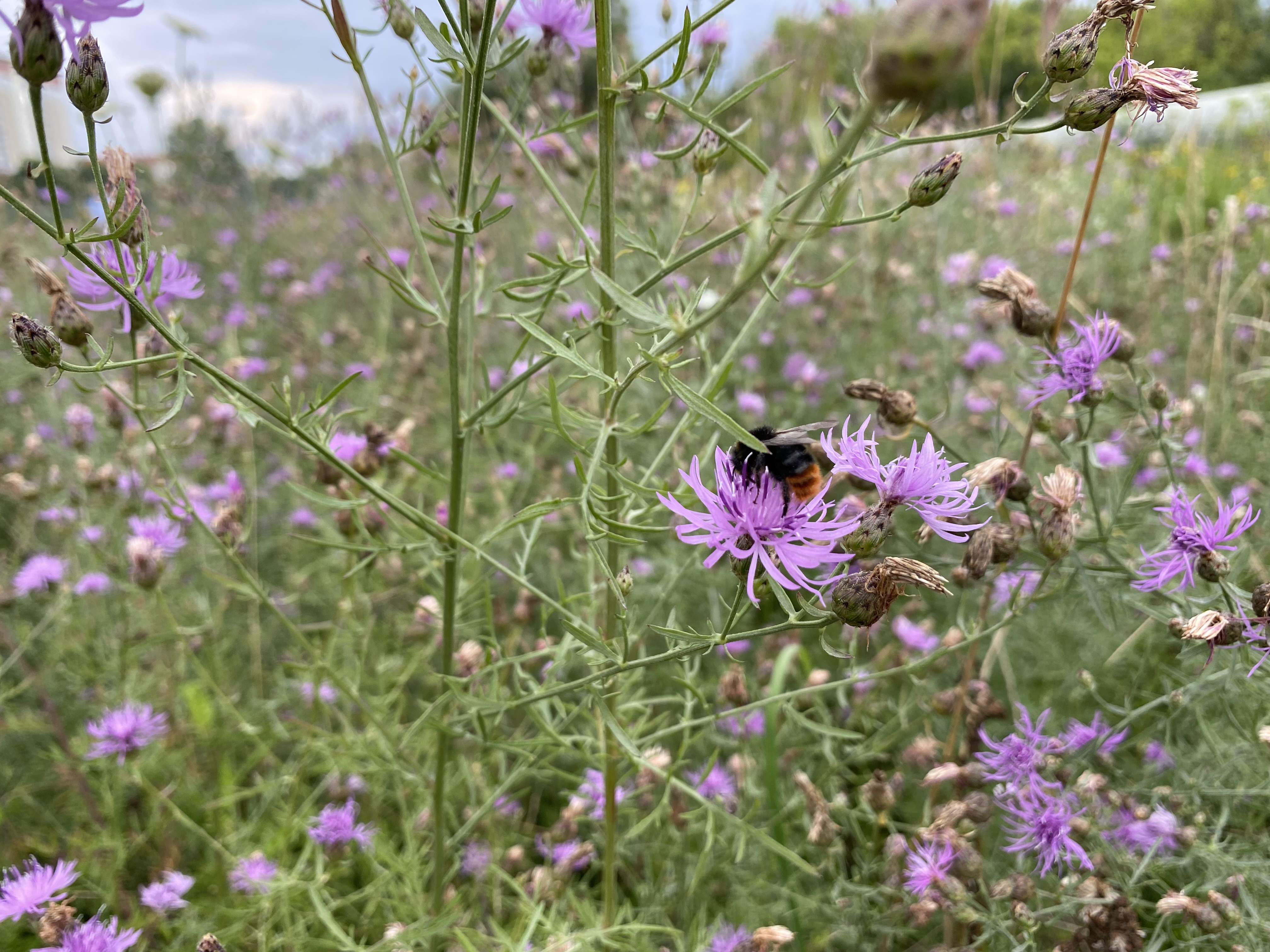

Supplement: Supplementary file 1 — Data S1. [file ECE3-15-e70791-s001.zip › Bombus lapidarius + Centaurea stoebe - Foto Anita J. Grossmann.jpg]
